# Genome-wide identification of the *COBRA-like* gene family and expression pattern analysis under abiotic stresses of *Sorghum bicolor* (L.)

**DOI:** 10.3389/fpls.2025.1652613

**Published:** 2025-09-09

**Authors:** Shipeng Liu, Shuang Liang, Tingrui Jing, Xinyi Guo, Hairuo Wang, Quan Ma, Junshen Wang, Kai Wang, Xiaolong He, Haibing Zhao, Wenting Jiang, Xiangqian Zhang

**Affiliations:** ^1^ College of Life Sciences, Yan’an University, Yan’an, China; ^2^ Engineering Research Center of Microbial Resources Development and Green Recycling, University of Shaanxi Province, Yan’an University, Yan’an, China

**Keywords:** *SbCBL* gene family, systematic evolution, whole genome duplication, abiotic stresses, *SbCBL4/9*

## Abstract

**Introduction:**

*COBRA-Like* (*CBL*) genes encode glycosylphosphatidylinositol (GPI) -anchored proteins specific to plants that play important roles in cellulose biosynthesis in primary and secondary cell walls.

**Methods:**

This study used a bioinformatics approach to characterize the *CBL* family genes in *Sorghum bicolor* (*S. bicolor*) at the genome-wide level to investigate their potential functions in *S. bicolor* development.

**Results:**

The results revealed the identification of 10 *CBL* genes in the BTx623 and E048 *S. bicolor* genomes, respectively. A comparative analysis of conserved Motifs revealed that all *CBL* family genes in *S. bicolor* possess CCVS conserved structural domains. Phylogenetic analysis revealed that the family can be divided into two subfamilies, with genes within each subfamily exhibiting similar gene structures and physicochemical properties. Whole Genome Duplication (WGD) played an important role in the expansion of *SbCBL* gene family. The tissue-specific expression patterns of *SbCBL* genes suggest varying expression levels across different organs and tissues in *S. bicolor*, with *SbCBL1*, *SbCBL5*, and *SbCBL9* showing significantly higher expression levels in roots. PEG and NaCl treatments significantly affected *SbCBL* expression levels. *SbCBL4* expression increased after PEG treatment, while *SbCBL9* expression decreased after NaCl treatment.

**Conclusions:**

Overall, this study provides new insights into the role of the *CBL* gene family in *S. bicolor*.

## 1 Introduction

The *COBRA-Like* (*CBL*) gene family encodes a class of glycosylphosphatidylinositol (GPI)-anchored proteins that act as cell surface receptors localized directly to the outer surface of plant plasma membranes and are involved in the perception and transduction of cell wall remodeling signals (Schindelman et al., 2001; Roudier et al., 2002; Ringli, 2010). *CBL* gene family was originally identified in mutants of *Arabidopsis thaliana* (*A. thaliana*) root cells that are abnormally expanded (Benfey et al., 1993). The number of members of the *CBL* gene family varies considerably among species: *A. thaliana*, *Oryza sativa* (*O. sativa*), *Zea mays* (*Z. mays*), and *Gossypium hirsutum* (*G. hirsutum*) contain 12, 11, 9, and 39 members, respectively (Roudier et al., 2002; Li et al., 2003; Brady et al., 2007; Fu et al., 2024). Typical structural features include an N-terminal signal peptide that mediates endoplasmic reticulum localization, an aromatic amino acid-rich region that constitutes the cellulose binding site, a central CCVS structural domain (containing cysteine clusters) that maintains protein conformation, and a C-terminal GPI-anchored signal sequence (containing a ω-site) that mediates membrane localization (Roudier et al., 2002).

The *cob* mutant in *A. thaliana* exhibits abnormal root cell expansion and a dwarfing phenotype (Roudier et al., 2005). *In situ* hybridization results showed that the *COB* gene is highly expressed in the root elongation region, suggesting its involvement in regulating cellulose deposition during cell elongation (Roudier et al., 2005). Further studies revealed that *COB* gene mutations disrupt the orientation of cellulose microfilament arrangement, triggering a decrease in cellulose content (Roudier et al., 2005). The *COBL4* mutant, homologous to *COB*, exhibited a significant decrease in secondary wall cellulose content (Brown et al., 2005). In addition to CBL, five other cloned *A. thaliana* family members (e.g., *AtCOBL4*, *AtCOBL9* and *AtCOBL2*) are involved in cell wall synthesis. The *AtCOBL4* affects secondary wall cellulose synthesis (Brown et al., 2005); the *AtCOBL9* mutation results in defective polar root hair growth (Jones et al., 2006); and the *AtCOBL2* is involved in seed coat cellulose deposition (Ben et al., 2015). Notably, complete deletion of the *COB* gene triggers plant growth arrest and aberrant expression of defense-related genes (Ko et al., 2006). In monocotyledonous plants, a mutant of the *O. sativa BC1* gene (which encodes a CBL protein) exhibits a “brittle rod” phenotype and has 60.7% amino acid sequence homology with the *A. thaliana* COB protein (Li et al., 2003). This mutation results in reduced cell wall thickness, decreased cellulose content, abnormal lignin deposition, and decreased mechanical strength (Li et al., 2003). Wu Lab identified the *OsBCL4* gene, which encodes a CBL protein containing a typical GPI structural domain. Its T-DNA insertion mutant exhibits cell expansion, cellulose reduction, and pectin accumulation (Dai et al., 2011). In addition, the *OsBCL5* mutation affects male gametophyte transport (Dai et al., 2009), OsBC5 is involved in secondary wall formation in stem nodes (Aohara et al., 2009), and OsBC6 encodes a CESA-related protein that affects secondary wall synthesis (Kotake et al., 2011). In maize, ZmBK2L3, a member of the ZmBK2L family, is closely related to AtCOB, and its encoded protein retains conserved structural domains and is widely expressed at different developmental stages (Brady et al., 2007; Cao et al., 2012). Recent studies have confirmed that ZmBK2L1 is involved in the regulation of root hair development (Hochholdinger et al., 2008). Studies have shown that COBL genes play a role in how plants respond to abiotic stresses. For instance, DROT1 encodes a COBL protein. It was discovered that DROT1 increases cellulose content and maintains cellulose crystallinity in rice. This modulates the cell wall structure and enhances the plant’s resistance to drought (Sun et al., 2022). In *A. thaliana*, overexpressing *PtCOBL12* promotes plant growth and increases cellulose content and relative crystallinity (Geng et al., 2023). It also improves growth under drought stress conditions. Additionally, the *GhCOBL22* gene plays a pivotal role in cotton’s response to drought stress (Fu et al., 2024).

*Sorghum bicolor* (*S. bicolor*), as the fifth largest cereal crop in the world, is an important energy and forage crop with excellent agronomic traits such as high photosynthetic efficiency, high nutritional value, high adaptability, and resistance to drought and salinity, and an important model crop for the study of other energy crops (Silva et al., 2022). BTx623 and E048 are two distinct sorghum varieties. BTx623 was the first variety for which a high-quality whole genome was sequenced and assembled (Paterson et al., 2009). Its genome has become a universal “reference template” for sorghum research, providing an important foundation for gene targeting and editing (e.g., CRISPR) as well as functional validation. E048, on the other hand, is derived from Sudanese tropical germplasm (Early Hegari) and differs significantly from BTx623 in terms of disease resistance gene clusters and metabolic pathways. These differences make E048 ideal for comparative genomics studies. For these reasons, BTx623 and E048 were selected for this study. In this study, we identified members of the *S. bicolor CBL* gene family at the genome-wide level using bioinformatics methods, analyzed their gene structures, evolutionary relationships, selective pressures, and expression patterns, and laid the groundwork for elucidating the functions of this gene family in the stress response of *S. bicolor*.

## 2 Materials and methods

### 2.1 Identification of gene family members

In this study, HMMER 3.3.2 software was used to perform a homology search (E-value threshold of 1e^-5^) based on the Hidden Markov Model (HMM) of the CBL domain (PF04833) in the Pfam database (https://pfam.xfam.org/) (Mistry et al., 2021) for *S. bicolor* BTx623 and E048 protein sequences downloaded from the Phytozome v13 (https://phytozome-next.jgi.doe.gov/) (Goodstein et al., 2012) and SGMD databases (https://S.bicolor.genetics.ac.cn/SGMD, accessed on 5 May 2025) (Chen et al., 2025), respectively. The gene sequences obtained from the initial screening were further validated for conserved structural domains using NCBI Conserved Domain Database (CDD) (https://www.ncbi.nlm.nih.gov/Structure/bwrpsb/bwrpsb.cgi) to ensure that the identified sequences contained complete and typical CBL domains, thus accurately identifying members of the *S. bicolor CBL* gene family.

### 2.2 Analysis of gene structure and conserved motifs

The coding sequences (CDS) of the genes were aligned with the corresponding genomic sequences and visualized using TBtools software (Chen et al., 2023) to show the exon-intron structure of the *SbCBL* genes. Then, the conserved motifs of *S. bicolor* CBL proteins were predicted using MEME Suite 5.5.3 (https://meme-suite.org/meme/tools/meme) (Bailey et al., 2009) software, setting the maximum number of motifs to 10, the motif length range from 6-50 amino acids, and other parameters as default. In addition, the conserved structural domains of the *S. bicolor* CBL gene family were predicted by NCBI-CDD. Finally, the gene structures, conserved motifs, and conserved structural domains of *S. bicolor CBL* gene family members were comprehensively analyzed using TBtools software (Chen et al., 2023).

### 2.3 Phylogenetic tree construction

The amino acid sequences of *S. bicolor CBL* gene family members and CBL protein sequences of *A. thaliana*, *O. sativa*, *Z. mays*, *Solanum lycopersicum* (*S. lycopersicum*) and *Setaria italica* (*S. italica*) were subjected to multiple sequence comparison using MEGA 7.0 software (Kumar et al., 2016). Neighbor-joining (NJ) was then used to construct the phylogenetic tree. In the parameter settings, the number of bootstrap tests was 1000, and the Poisson correction model was selected to calculate the genetic distance. Finally, the evolutionary tree was embellished by iTOL (https://itol.embl.de/) online website to show the evolutionary relationship between *S. bicolor CBL* gene family and members of this gene family in other plants, and to analyze the evolutionary pattern and classification of the gene family.

### 2.4 Analysis of replication events and selection pressure

Tandem and genome-wide replication events of the *S. bicolor* CBL gene family were analyzed using MCScan X (Wang et al., 2012). The Simple Ka/Ks Calculator module in TBtools software was used to input the coding sequences (CDS), protein sequences, and immediately homologous gene pairs of the genes, respectively, and the Ka (non-synonymous substitution rate)/Ks (synonymous substitution rate) values between homologous genes were calculated to estimate the selection pressure. In addition, MCScan X (Wang et al., 2012) was applied to analyze the covariation events between *S. bicolor* and *A. thaliana*, *S. lycopersicum*, *O. sativa*, and *S. italica CBL* gene families.

### 2.5 Gene expression analysis

Transcriptome data of different tissues of *S. bicolor* (including seedlings, leaves, roots, stems, inflorescences, and seeds, etc.) at different developmental stages were obtained from the *S. bicolor* Genome and Mutant Bank SGMD database (Chen et al., 2025). Then, we utilized Tbtools software to analyze gene expression data from various tissues, screening for CBL genes that exhibited high expression levels across different tissues and developmental stages. The data are shown in a heatmap with gene expression in different tissues with row-scaled transcriptome atlas (TPM values). Red and blue boxes indicate high and low expression levels of *SbCBL* genes.

### 2.6 RT-qPCR

*S. bicolor* was grown in a growth chamber at Yan’an University with 16.0 hours of light, temperature maintained at 25°C and 70% humidity. To determine the expression level of *CBL* gene after NaCl and PEG treatments, *S. bicolor* seedlings at the three-leaf-one-heart stage were selected and treated with 150 mM NaCl and 15% PEG, respectively, followed by collection of *S. bicolor* root samples 7 days. All experiments were performed in three biological replicates (Three biological replicates and three technical replicates per biological sample were performed.). Total RNA was isolated using the Plant Total RNA Kit from Beijing Zhuangmeng International BioGenetics Co. Ltd. and reverse transcription was performed using HiScript IV All-in-One Ultra RT SuperMix for qPCR from Novozymes. The RT-qPCR amplification reaction system consisted of 5 μL 2×SYBR, 3 μL ddH_2_O, 1 μL cDNA template, and 0.5 μM forward and reverse primers in a total volume of 10 μL. The expression level of *SbCBL* gene was analyzed by the 2^-ΔΔCT^ method in response to different stress treatments (Xue et al., 2024), and *SbACTIN* was used as an internal reference gene to analyze the expression level of *SbCBL* gene under different stress treatments.

## 3 Result

### 3.1 Identification of *S. bicolor CBL* gene family members

**Figure 1:**
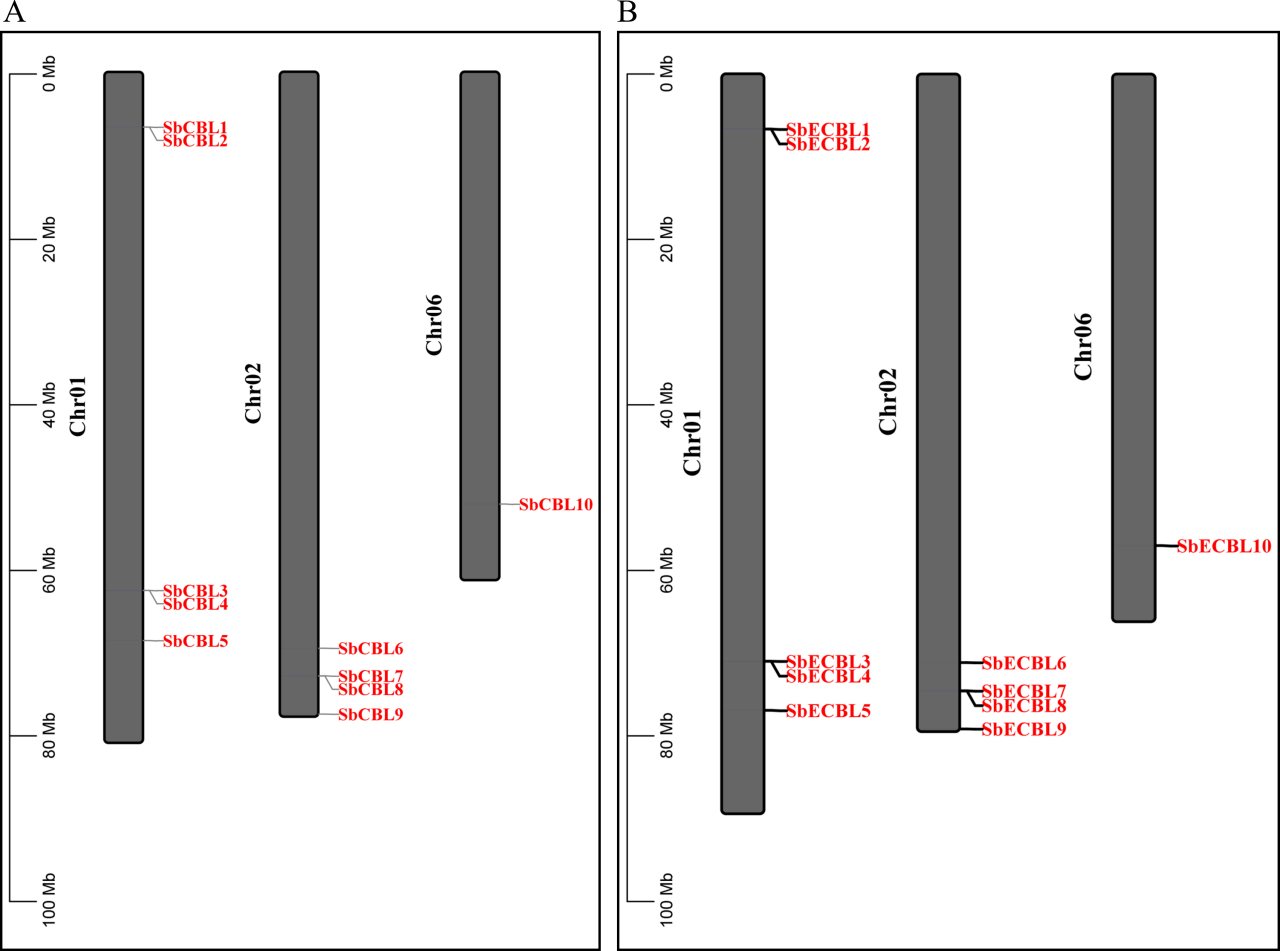
Chromosomal distribution of *CBL* genes in *S. bicolor*. **(A)** Chromosomal distribution of *CBL* genes in *S. bicolor* BTx623. **(B)** Chromosomal distribution of *CBL* genes in *S. bicolor* E048.

Using HMMER 3.3.2 software based on the Hidden Markov Model of the CBL conservative domain (PF04833) in the Pfam database, we searched and validated *S. bicolor* protein sequences using NCBI-CDD. Finally, we identified ten *CBL* gene family members. These genes were named *SbCBL1*-*SbCBL10* and *SbECBL1*-*SbECBL10* based on their location on the chromosomes. The distribution of these 20 genes on the *S. bicolor* chromosomes was visualized using TBtools software, and the results are shown in **Figure 1**. *S. bicolor CBL* gene family members were unevenly distributed across three chromosomes: five genes (*SbCBL1*-*SbCBL5*) were found on chromosome 1; four genes were found on chromosome 2; and one gene was found on chromosome 6. This uneven distribution pattern may be related to the evolution of gene families, chromosome structure, and gene function.

**Figure 2:**
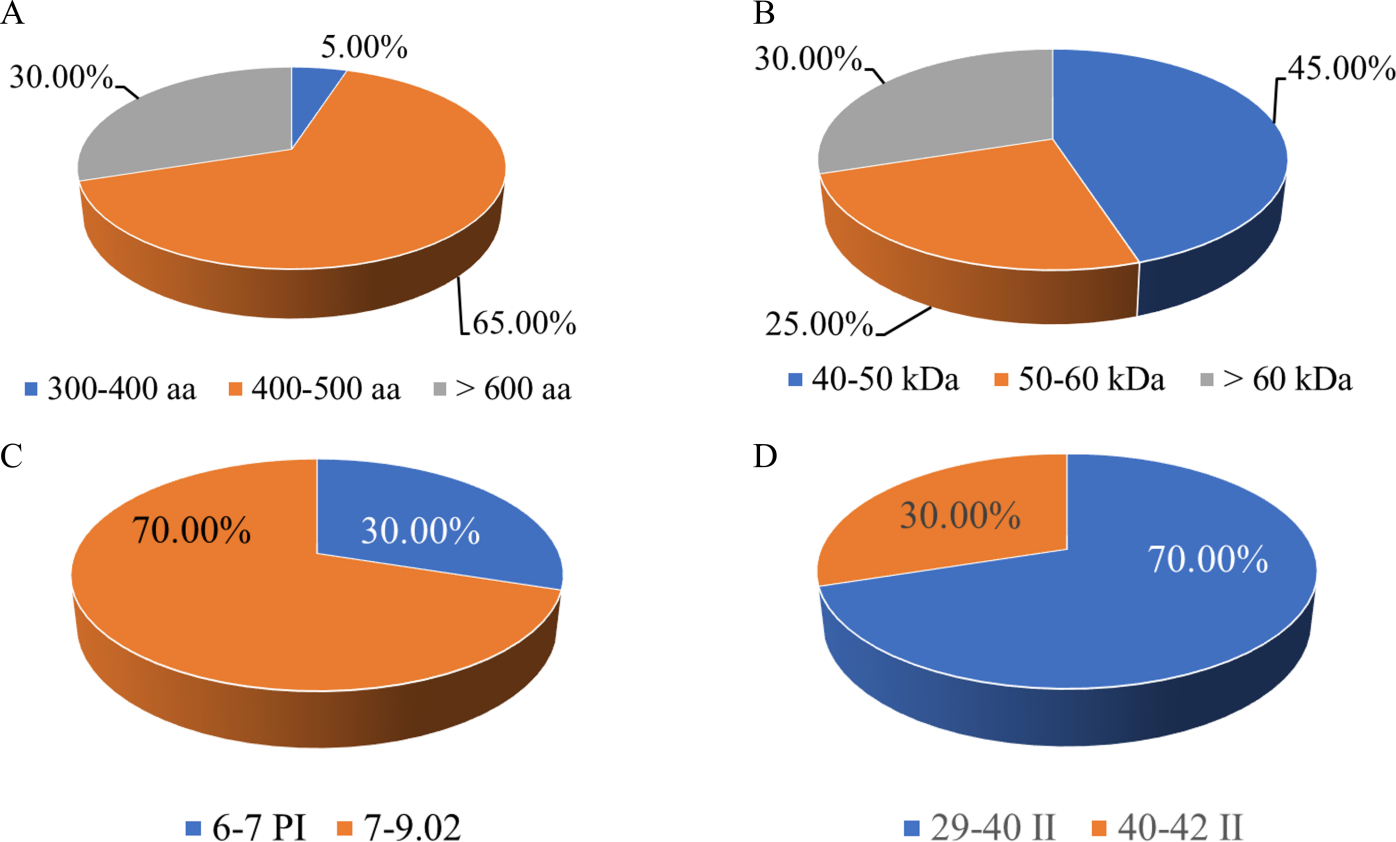
Analysis of physicochemical properties of *S. bicolor* CBL protein. **(A)** Statistics of amino acid length of *S. bicolor* CBL protein. **(B)** Statistics of molecular weights of *S. bicolor* CBL protein. **(C)** Statistics of Isoelectric point of *S. bicolor* CBL protein. **(D)** Statistics of Instability Index of *S. bicolor* CBL protein.

This study systematically analyzed the amino acid length, molecular weight, isoelectric point, instability index, and hydrophilicity of these members. The results showed that the lengths of the amino acid sequences encoded by the *S. bicolor* CBL gene family members differed significantly. For instance, SbCBL1 encodes 187 amino acids, whereas SbCBL7 encodes a protein consisting of 673 amino acids (**Figure 2A**). Additionally, the amino acid lengths of CBL homologous genes differed between the two *S. bicolor* varieties. For instance, SbCBL9 corresponds to SbECBL9, which has 446 and 686 amino acids, respectively. This difference in length may reflect the functional complexity of different members. In terms of molecular weight, members of the *S. bicolor* CBL gene family exhibited similar diversity. The results showed that family proteins have a wide range of molecular weights. For example, SbCBL1 has a molecular weight of 46.832 kDa, while SbCBL7 has a molecular weight as high as 74.62 kDa. There are also members with a predicted molecular weight of about 36.72 kDa (**Figure 2B**). This difference in molecular weight may be closely related to the structural and functional diversity of the proteins. Notably, 70% of the *SbCBL* gene family members in Sorghum bicolor exhibit an isoelectric point (pI) exceeding 7. This prevalence of basic pI values underscores the potential functional adaptations of these proteins for operating in alkaline cellular environments or interacting with negatively charged macromolecules (**Figure 2C**). Additionally, 70% of SbCBL proteins were predicted to be unstable (PI ≥ 40), while 30% were stable (**Figure 2D**). This result establishes a foundation for the subsequent in-depth study of this family’s protein functions. Through computational predictions of subcellular localization, all members of the SbCBL protein family exhibited exclusive targeting to the plasma membrane, indicating a highly conserved localization pattern. This striking uniformity in membrane association strongly suggests that SbCOBL proteins may play specialized roles in cell wall-plasma membrane interactions, potentially regulating cellulose deposition patterns or mediating mechanical stress responses at the cell surface.

### 3.2 Systematic evolutionary analysis

**Figure 3:**
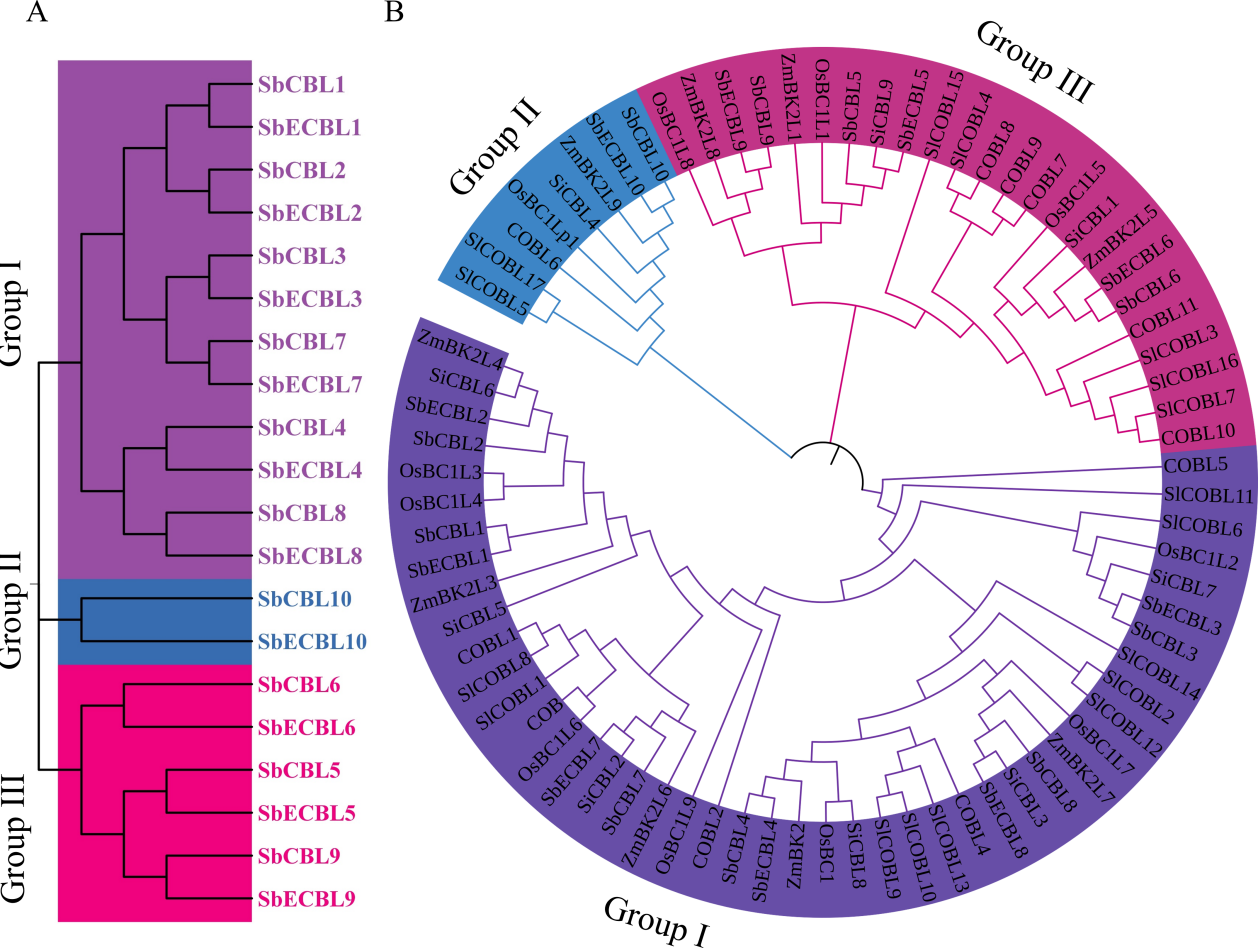
Phylogenetic analysis of CBL proteins. **(A)** Phylogenetic analysis of CBL proteins in *S. bicolor*. The evolutionary tree was constructed using the neighbor-joining (NJ) method with 1,000 bootstrap replicates in MEGA 7. CBL proteins were classified into three subfamilies: Group I, Group II, and Group III, represented by purple, blue and pink branches, respectively. **(B)** Phylogenetic analysis of CBL proteins in *S. bicolor*, *A thaliana*, *S. lycopersicum*, *O. sativa*, *S. italica* and *Z. mays*. The evolutionary tree was constructed using the neighbor-joining (NJ) method with 1,000 bootstrap replicates in MEGA 7. CBL proteins were classified into three subfamilies: Group I, Group II, and Group III, represented by purple, blue and pink branches, respectively.

To resolve the evolutionary classification of the *S. bicolor CBL* gene family, this study used MEGA 7.0 software to conduct a phylogenetic analysis of the amino acid sequences of 10 SbCBL and 10 SbECBL members. The results showed that the CBL proteins of the two *S. bicolor* varieties can be categorized into three subfamilies: Group I, Group II, and Group III (**Figure 3A**). Group I contains 12 members: SbCBL1, SbCBL2, SbCBL3, SbCBL4, SbCBL7, SbCBL8, SbECBL1, SbECBL2, SbECBL3, SbECBL4, SbECBL7, and SbECBL8. Group II includes SbCBL10 and SbECBL10, and Group III includes SbCBL5, SbCBL6, SbCBL9, SbECBL5, SbECBL6, and SbECBL9.

**Figure 4:**
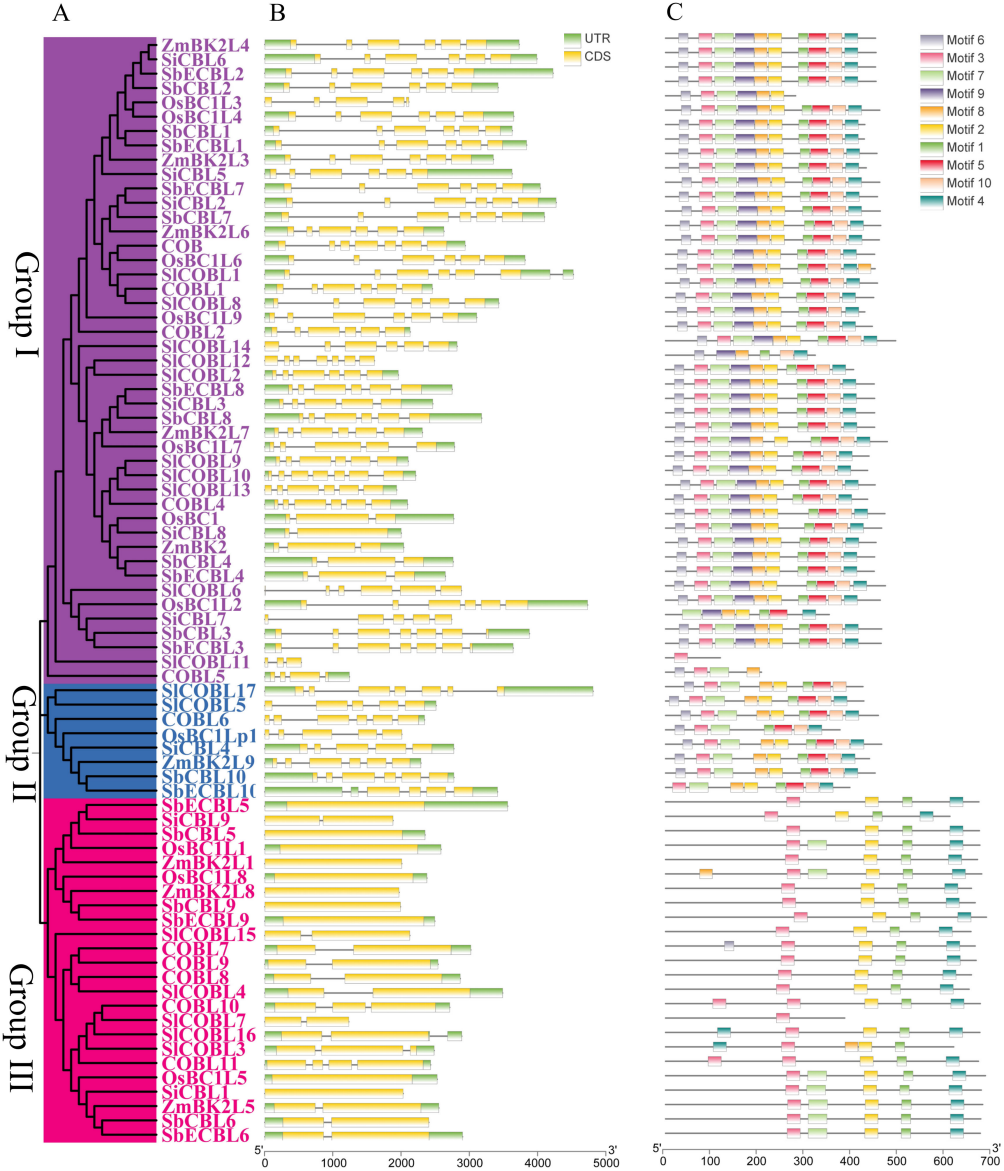
Phylogenetic, conserved motif, domain, and gene structure analysis of *S. bicolor*, *A thaliana*, *S. lycopersicum*, *O. sativa*, *S. italica* and *Z. mays* CBL proteins. **(A)** Phylogenetic analysis of *S. bicolor*, *A thaliana*, *S. lycopersicum*, *O. sativa*, *S. italica* and *Z. mays* CBL proteins. The neighbor-joining (NJ) tree was constructed using MEGA 7 with 1,000 bootstrap replicates. **(B)** Intron-exon structure of *S. bicolor*, *A. thaliana*, *S. lycopersicum*, *O. sativa*, *S. italica* and *Z. mays CBL* genes. Visualization was performed using TBtools. **(C)** Conserved motif analysis of *S. bicolor*, *A. thaliana*, *S. lycopersicum*, *O. sativa*, *S. italica* and *Z. mays CBL* proteins. Ten motifs were identified using the online tool MEME with default parameters.

To further elucidate the phylogenetic position of the *S. bicolor CBL* genes, this study integrated CBL protein sequences from *S. bicolor*, *A. thaliana*, *S. lycopersicum*, *O. sativa*, *S. italica* and *Z. mays* for a multiple comparison analysis (**Figure 3B**). Phylogenetic trees constructed using the neighbor-joining method showed that the *CBL* genes of all species could be divided into three significant branches (**Figure 4B**). Group I mainly included six SbCBLs, six SbECBLs, five AtCBLs, ten SlCBLs, seven OsCBLs, five ZmCBLs, and six SiCBLs. Group II mainly consisted of three SbCBLs, three SbECBLs, five AtCBLs, five SlCBLs, three OsCBLs, three ZmCBLs, and two SiCBLs. Group III mainly consisted of one SbCBL, one SbECBL, one AtCBL, two SlCBLs, one OsCBL, one ZmCBL, and one SiCBL. The results of the phylogenetic analysis showed that the *S. bicolor CBL* gene family has evolved to be related to *CBL* genes from other plants while maintaining its own specificity. ZmBk2L3, a COBRA family protein, functions in the regulation of cell wall dynamics and carbohydrate partitioning (Julius et al., 2021). Phylogenetic analysis revealed that its clustering with SbCBL1/2 and SbECBL1/2 suggests involvement in cell membrane-associated signaling or cell wall modification via similar mechanisms. ZmBk2 maintains the flexibility of plant organs by modulating the lignin-cellulose interaction pattern (Sindhu et al., 2007). Additionally, phylogenetic analyses revealed that ZmBk2 clusters with SbECBL4 and SbCBL4, indicating that the protein may perform similar functions via conserved molecular mechanisms.

### 3.3 Gene structure and conserved motif characterization

A gene structure and conserved Motif analysis of *S. bicolor CBL* genes revealed significant structural and functional differences. Gene structure revealed notable variations in exon-intron structure among different subgroups. Specifically, members of subgroup I have three to seven exons and exhibit structural diversity. Members of subgroup II, such as SbCBL10 and SbECBL10, have six and four exons, respectively. Notably, subgroup III members have a more concise gene structure with only one or two exons. This diversity in gene structure may be closely related to functional differentiation and the evolutionary history of genes (**Figures 4A, B**). Different exon-intron structures may lead to variations in gene transcription and translation processes, which may affect gene function and expression regulation.

Conserved motif analysis performed by MEME Suite 5.5.3 identified a total of 10 characteristic motifs (Motif1-Motif10). Systematic analysis revealed that all motifs were intact in subgroup I, suggesting that these core elements may collectively maintain the basal biological functions of CBL proteins. Subgroup II exhibited a distinct motif deletion pattern: SbCOBL10 lacked Motif9, and SbECOBL10 lacked Motifs 1 and 9 (**Figure 4C**). Members of the third subgroup contained only Motifs 1, 2, 3, and 4, which were distributed in a manner that may be related to the functional differentiation of gene family members. The distribution of motifs 1, 2, 3, and 4 is specific and may be related to the functional differentiation of gene family members (**Figure 4C**). Analysis of gene structure and conserved motifs revealed that *S. bicolor CBL* gene family members are structurally conserved yet diverse. These conserved structural features may ensure the gene family’s basic function, while diverse structures provide the basis for the genes’ functional differentiation and evolution, enabling different gene members to play unique roles in *S. bicolor* growth, development, and environmental adaptation.

### 3.4 Analysis of duplication events

**Figure 5:**
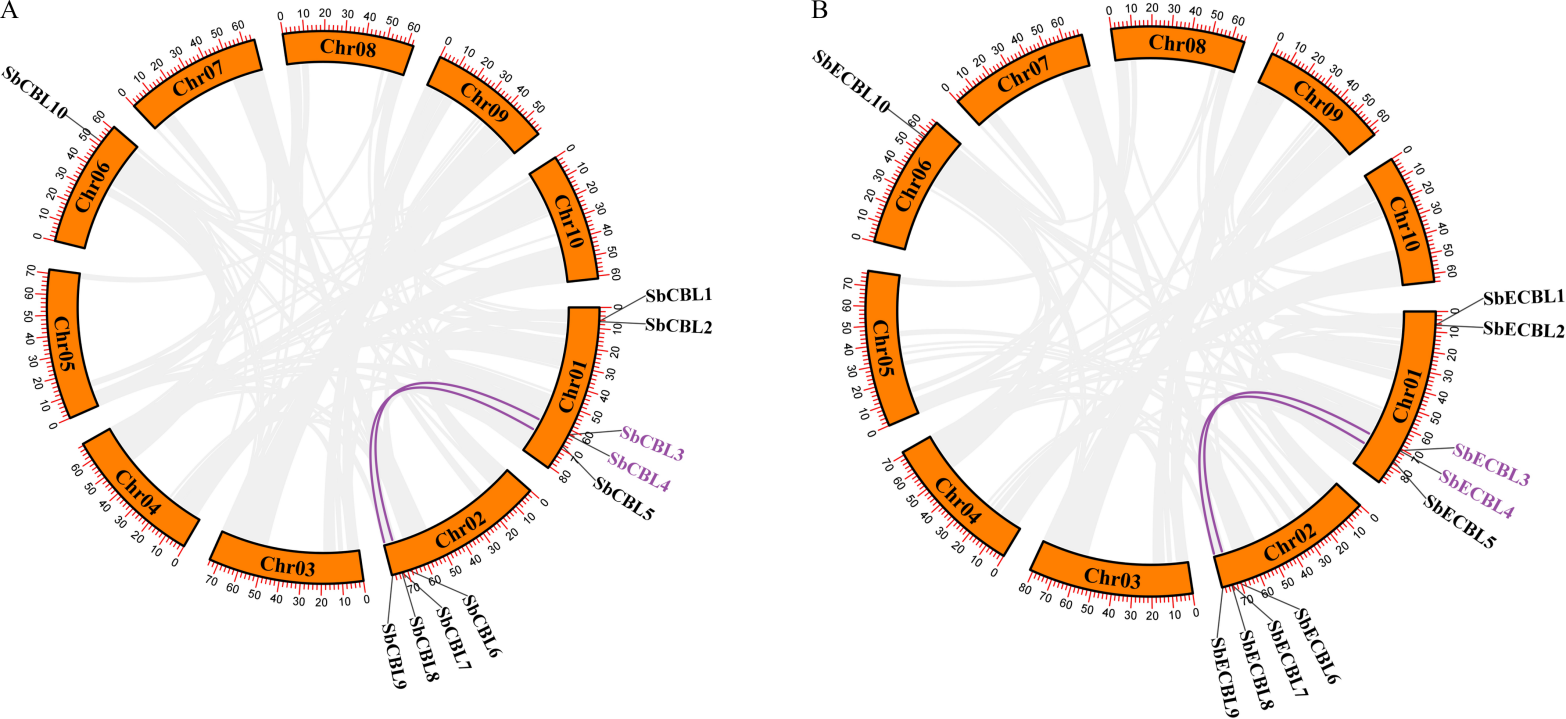
Analysis of gene duplication events of *CBL* genes in *S. bicolor*. **(A)** Gene duplication events of *S. bicolor* BTx623 *CBL* genes. Whole-genome duplication (WGD) events are indicated by purple lines, and tandem duplicated genes are labeled with purple gene IDs. **(B)** Gene duplication events of *S. bicolor* E048 *CBL* genes. Whole-genome duplication (WGD) events are indicated by purple lines, and tandem duplicated genes are labeled with purple gene IDs.

To reveal the expansion mechanism of the *S. bicolor CBL* gene family and its evolutionary constraints, this study systematically analyzed the types of replication events and selection pressures that characterize its members. As shown in the **Figure 5**, tandem duplication (TD) events drove the clustered distribution of CBL3 and CBL4. Meanwhile, Whole Genome Duplication (WGD) events contributed to the generation of two paralogous gene pairs: CBL3/CBL7 and CBL5/CBL9.

Further analysis of the ratio of non-synonymous to synonymous substitution rates (Ka/Ks) revealed that the Ka/Ks values of these replication events were significantly less than one (CBL3/CBL7: Ka/Ks = 0.1; CBL5/CBL9: Ka/Ks = 0.27), suggesting that these genes underwent strong purifying selection (**Supplementary Table 2**). This indicates that these genes experienced strong purifying selection during evolution.

### 3.5 Synteny analysis of *S. bicolor* and other species

**Figure 6:**
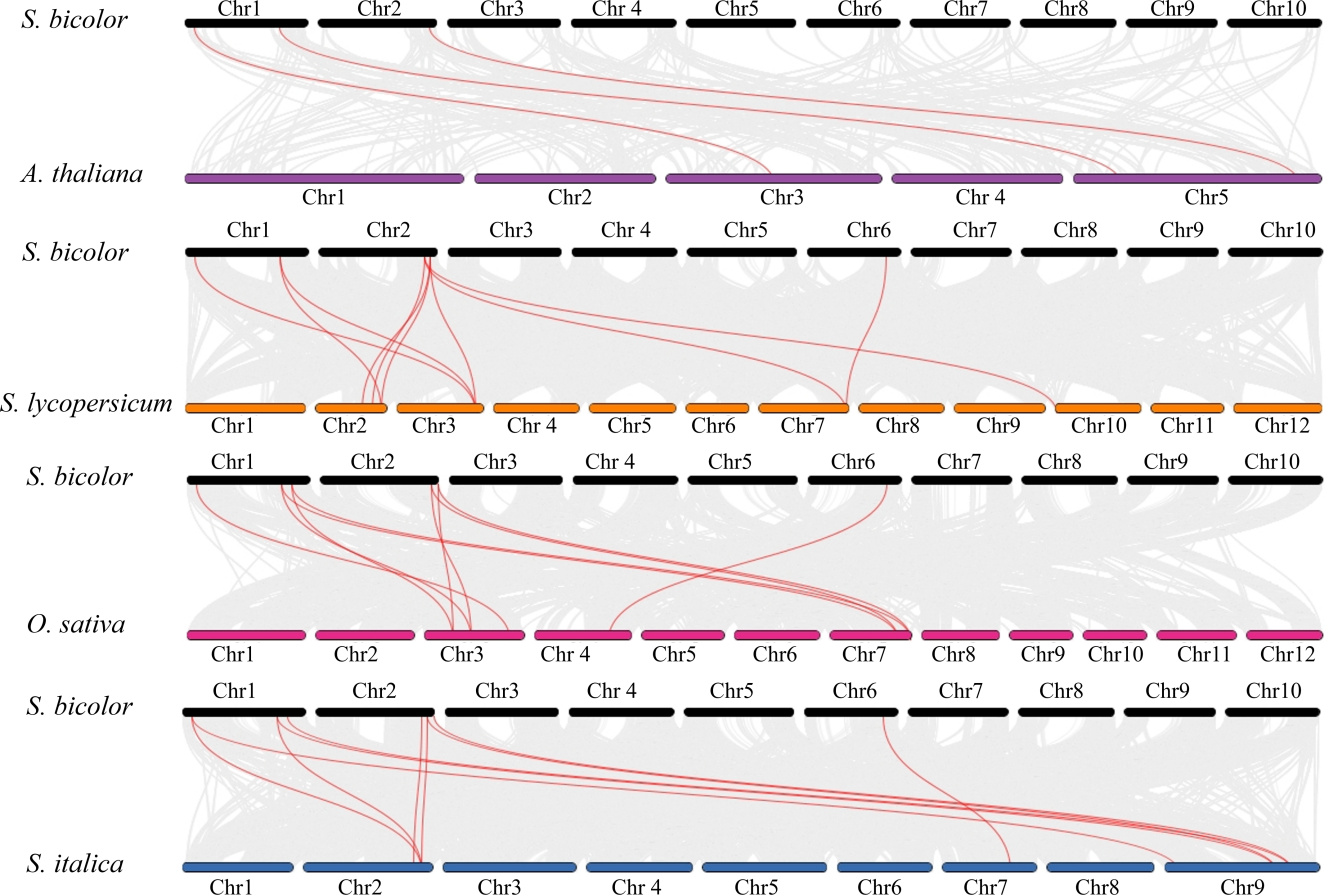
Synteny analysis of *CBL* genes with other species. Synteny between *S. bicolor* BTx623 and *A. thaliana CBL* genes. Synteny between *S. bicolor* and *S. lycopersicum CBL* genes. Synteny between *S. bicolor* and *O. sativa CBL* genes. Synteny between *S. bicolor* and *S. italica CBL* genes.

To elucidate the evolutionary trajectory of the *CBL* gene family in *S. bicolor*, this study performed whole-genome synteny analysis using MCScan X between S. bicolor and representative species, including the dicot model plant *A. thaliana*, monocot crops *O. sativa* and *S. italica*, as well as the solanaceous crop *S. lycopersicum*. A total of 3 (*S. bicolor*–*A. thaliana*), 10 (*S. bicolor*–*S. lycopersicum*), 10 (*S. bicolor*–*O. sativa*), and 10 (*S. bicolor*–*S. italica*) *CBL* homologous gene pairs were identified (**Figure 6**). Notably, *O. sativa*, *S. italica* and *S. bicolor*, as closely related species within the Gramineae family, exhibited significantly more syntenic gene pairs than *A. thaliana*, indicating that the *CBL* gene family retained higher genomic structural conservation after monocot–dicot divergence. Further analysis revealed “one-to-many” homologous relationships between certain *S. bicolor CBL* genes and multiple species. For instance, *SbCBL3* showed synteny with both *SlCBL1* and *SlCBL1* in *S. lycopersicum*. Similarly, *SbCBL3* corresponded to three homologs in *S. italica* (*SiCBL2* and *SiCBL7*) and two in *O. sativa* (*OsCBL1* and *OsCBL6*).

### 3.6 Analysis of promoter cis-acting elements of the *S. bicolor CBL* gene family

**Figure 7:**
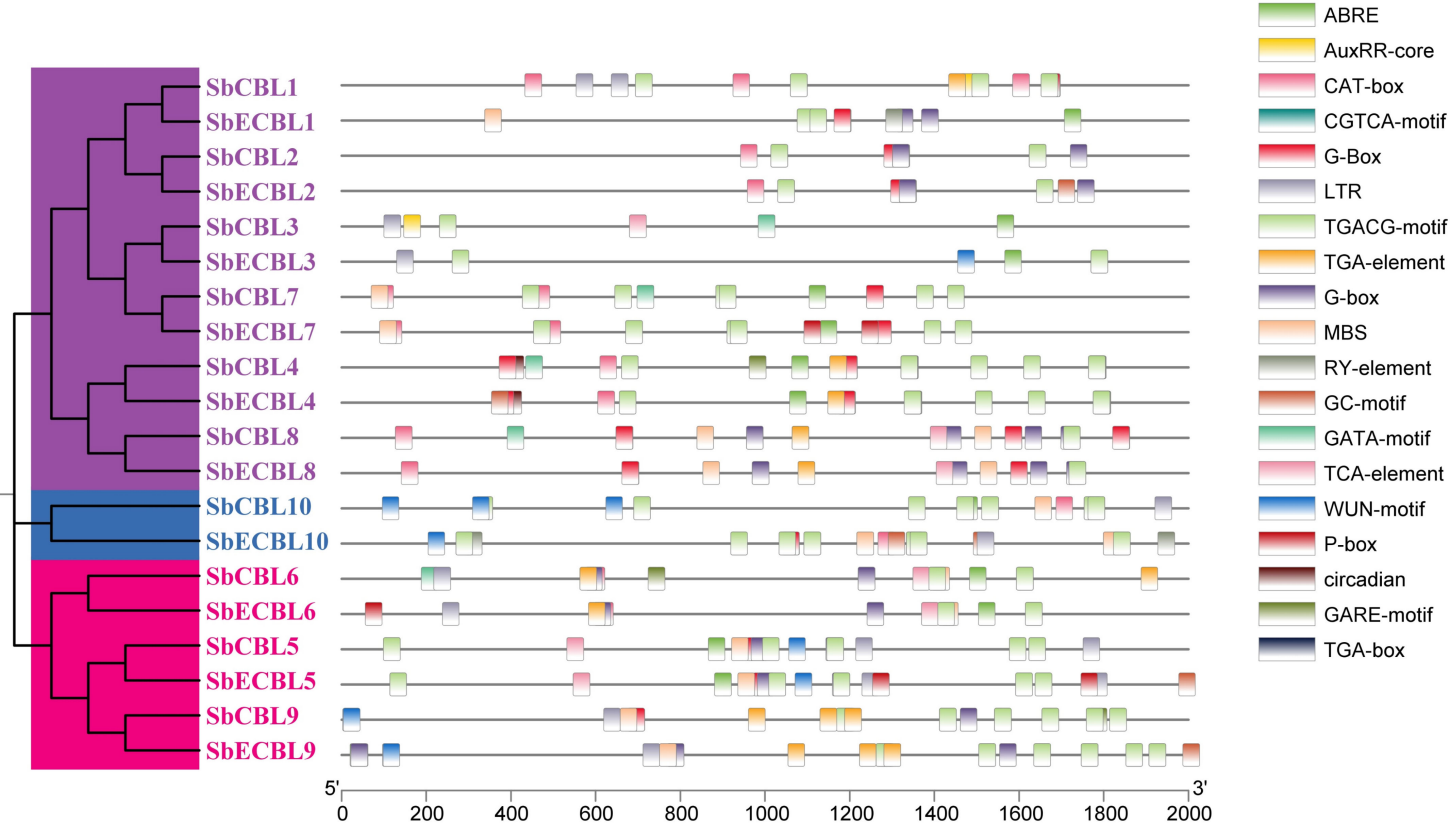
Analysis of cis-regulatory elements in the promoter regions of *S. bicolor CBL* gene.

To analyze the transcriptional and regulatory features of the *S. bicolor CBL* gene family, this study systematically analyzed the cis-acting elements in the upstream promoter region (2,000 bp prior to the transcriptional start site) of the gene using the PlantCARE database. The results showed that the *CBL* gene promoter region contained abundant regulatory elements, mainly categorized into four groups: hormone response, abiotic stress response, light signaling regulation, and growth and development (**Figure 7**; **Supplementary Table 3**). All members carried abscisic acid (ABA) response elements (ABRE, ACGTG) and methyl jasmonate (MeJA) response elements (TGACG motif, TGACG), indicating that the *S. bicolor CBL* gene family may be involved in regulating adversity acclimatization through ABA and MeJA signaling pathways. Additionally, some members contained cis-regulatory elements related to growth hormones, salicylic acid, and gibberellin, implying that these members may be involved in multiple stress responses through hormone crosstalk. Abiotic stress response elements included the drought response element MBS (CAACTG), the low temperature response element LTR (CCGAAA), the anaerobic-induced element ARE (AAACCA), and the mechanical damage response element. Members of subgroup III (e.g., *SbCBL7/9*) showed a notably high frequency of the low-temperature response element LTR (CCGAAA) in the promoter region. This density was significantly higher than that of the other subgroups. This suggests that subgroup III may enhance cold hardiness by activating low-temperature acclimation pathways. Meanwhile, subgroups II and III specifically carried the mechanical damage response element, the WUN motif (AAATTACCT), which may respond to physical stress by regulating the cell wall.

### 3.7 Analysis of gene expression patterns

**Figure 8:**
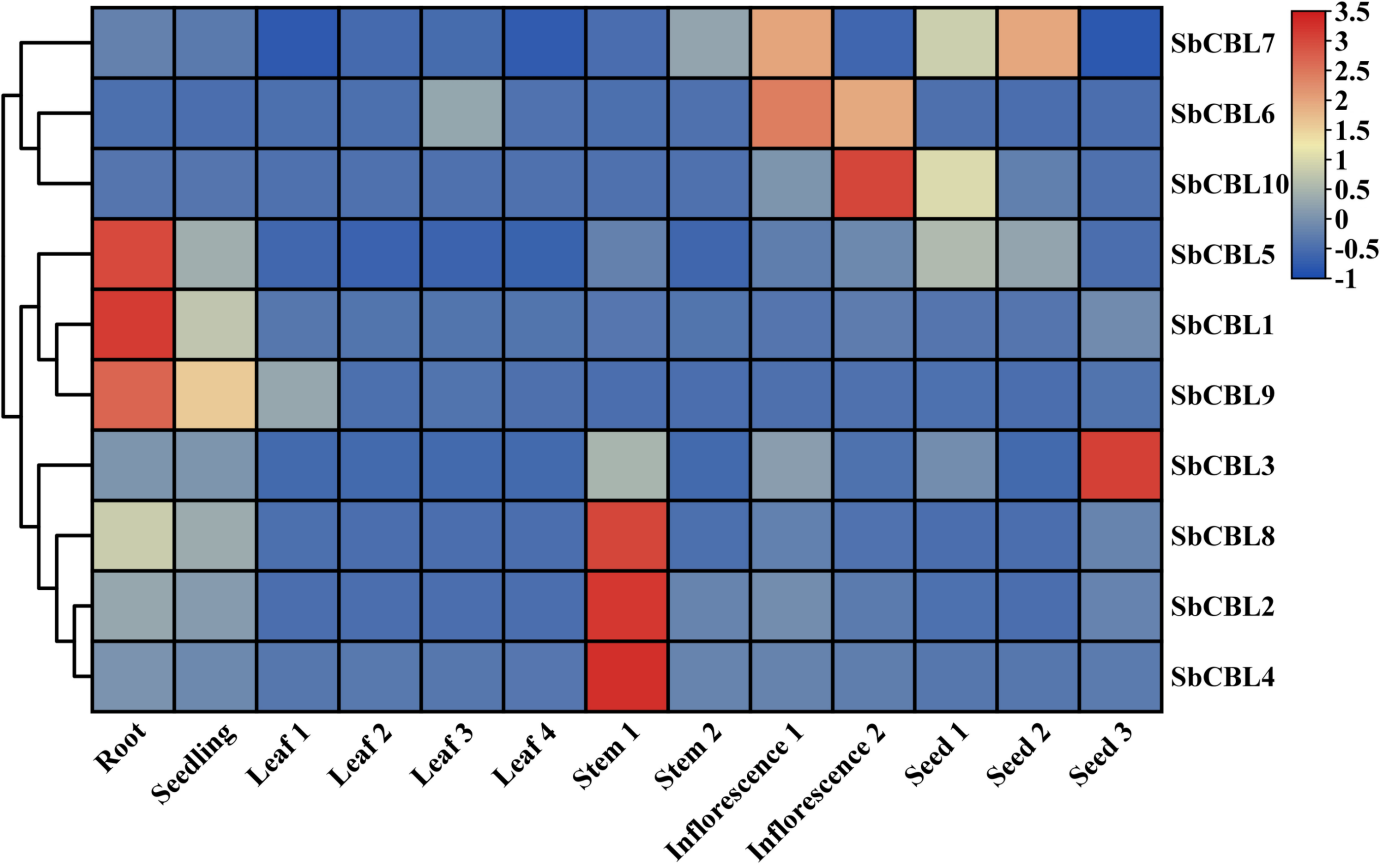
The heat map shows the expression level of the *S. bicolor CBL* gene in different tissues. Red and blue boxes indicate high and low expression levels of *SbCBL* genes.

We analyzed the expression patterns of 10 *S. bicolor CBL* gene family members based on transcriptome data of different *S. bicolor* tissues (e.g., roots, stems, leaves, flowers, and seeds) at different developmental stages in the SGMD database (**Supplementary Table 4**). The data are presented in a heatmap illustrating gene expression across various tissues, utilizing a row-scaled transcriptome atlas based on TPM (Transcripts Per Million) values. In this visualization, red boxes signify high expression levels of *SbCBL* genes, while blue boxes indicate low expression levels. As illustrated in **Figure 8**, various *CBL* genes exhibited significant expression variations across different *S. bicolor* tissues and developmental stages. In root tissues, the expression levels of *SbCBL1*, *SbCBL5* and *SbCBL9* were relatively high, suggesting that these genes play important roles in root growth and development. The normal development of roots is crucial for plant growth and survival because they are an important organ for water and nutrient uptake in plants. These genes may promote root growth and development by regulating cell wall synthesis and modification in root cells, as well as affecting cell elongation and differentiation. In stem tissues, *SbCBL2*, *SbCBL4* and *SbCBL8* exhibited high expression levels, suggesting their involvement in stem elongation and thickening processes. Stem growth and development play a key role in supporting the plant and transporting materials. These genes may regulate the arrangement and deposition of cellulose microfilaments in stems, enhancing their mechanical strength to support the plant during growth. *SbCBL6*, *SbCBL7* and *SbCBL10* are expressed at significantly higher levels in inflorescence tissues than in other tissues, suggesting that they play important roles in flower development and reproduction.

The expression level of *SbCBL3* was significantly higher in the seed grain than in other tissues, suggesting that this gene plays an important role in seed grain development. Because material accumulation and maturation directly affect *S. bicolor* yield and quality, *SbCBL3* may influence seed grain size, shape, and starch accumulation by regulating cell wall synthesis and modification, ultimately affecting *S. bicolor* yield and quality. Gene expression pattern analysis revealed that members of the *S. bicolor* CBL gene family exhibit distinct expression patterns in various tissues and developmental stages. This differential expression may be closely related to the functional differentiation of these genes. These gene family members play unique roles in different stages of *S. bicolor* growth and development, as well as in different tissues. They are involved in plant growth, development, and reproduction by regulating cell wall synthesis and modification.

### 3.8 Expression profile of *SbCBL* in *S. bicolor* in response to NaCl and PEG

**Figure 9:**
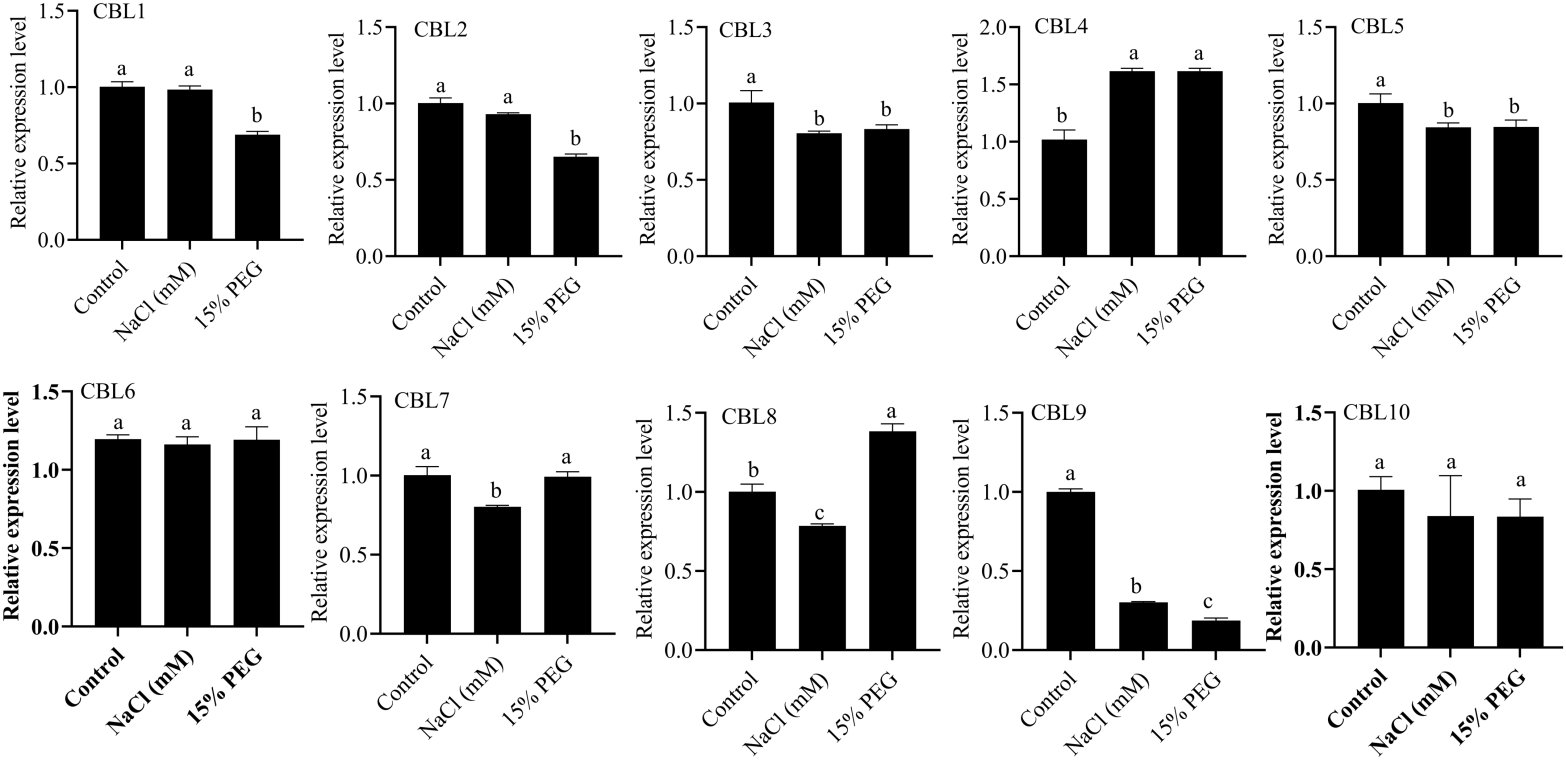
Analysis of the expression levels of *CBL1*, *CBL2*, *CBL3*, *CBL4*, *CBL5*, *CBL6*, *CBL7*, *CBL8, CBL9* and *CBL10* after treatment with NaCl and PEG. All data are means ± sd ( n ≥ 3). Letters a, b and c represent statistical significance, P < 0.05.

To explore the expression pattern of the *CBL* gene in *S. bicolor* under drought and salt stress, we selected a *CBL* gene with high expression levels in the roots and performed RT-qPCR analysis. As shown in **Figure 9**, the expression levels of several *CBL* genes changed significantly under NaCl and PEG treatment. Under NaCl treatment, the expression level of *SbCBL4* increased, while the expression levels of *SbCBL3*, *SbCBL5*, *SbCBL7*, *SbCBL8* and *SbCBL9* decreased. Conversely, PEG treatment up-regulated the expression of genes such as *SbCBL4* and *SbCBL8*, while down-regulating the expression of *SbCBL1*, *SbCBL2*, *SbCBL3*, *SbCBL5* and *SbCBL9*. Notably, the *SbCBL3*, *SbCBL5* and *SbCBL9* genes were down-regulated following both drought and salt stress treatments. These results suggest that the *CBL* gene in *S. bicolor* plays a role in the *S. bicolor* response to salt and drought stress.

## 4 Discussion

### 4.1 Structural and functional speculation of the CBL gene family in *S. bicolor*

In this study, 10 members of the *CBL* gene family were identified from the *S. bicolor* BTx623 and E048 genomes, respectively (**Figure 1**). Gene structure analysis showed that the number of exons of these members ranged from 1-7, and there were differences in exon lengths and intron phases (**Figure 3**). Through conserved motif analysis, we identified 10 conserved motifs, of which Motif1, Motif2, Motif3 and Motif4 were distributed among all members, indicating that these motifs are important for maintaining the basic structure and function of CBL proteins (**Figure 3**). The structural features of genes are usually closely related to their functions (Sajjad et al., 2023). The diversity in exon-intron structure of *S. bicolor CBL* gene family members implies that they may be functionally differentiated. For example, *SbCBL2*, *SbCBL3*, *SbCBL4*, *SbCBL7* and *SbCBL8*, which have a higher number of exons, may encode proteins with more complex structures and functions, which are involved in the regulation of multiple processes in *S. bicolor* growth and development, whereas *SbCBL5*, *SbCBL6* and *SbCBL9*, which have a relatively low number of exons, may encode proteins that are simpler. This phenomenon has been observed in other species (Sajjad et al., 2023). Based on gene structure and conserved motif analyses combined with existing research reports, it is hypothesized that members of the *S. bicolor CBL* gene family are involved in several aspects of *S. bicolor* growth and development. However, these functional speculations require further experimental validation. Future studies could knock out or overexpress members of the *S. bicolor CBL* gene family using gene editing techniques, such as CRISPR-Cas9, to observe their effects on growth and development, cell wall structure, and related physiological processes. Simultaneously, proteomics and biochemical methods will be used to thoroughly study the interaction mechanism between CBL proteins and other molecules and clarify their specific functions and pathways of action within the cell.

### 4.2 Comparison with *CBL* gene families of other species

In this study, we identified a total of 10 *CBL* genes in *S. bicolor*, which aligns closely with the number of genes in this family reported in other monocotyledonous plants. For instance, *O. sativa* has 11 *CBL* genes (Li et al., 2003), *Z. mays* has 9 (Brady et al., 2007). In contrast, among dicotyledonous plants, *A. thaliana* possesses 12 *CBL* genes (Roudier et al., 2002), *S. lycopersicum* has 17 (Cao et al., 2012), *P. trichocarpa* 14 (Sajjad et al., 2023), and *G. hirsutum* has 39. This indicates a significant variation in the number of *CBL* family genes across different plant species.This suggests that the *CBL* gene family is highly conserved across monocotyledonous. Phylogenetic tree analysis revealed that the *CBL* genes of *S. bicolor* are interspersed with those of *O. sativa* and *Z. mays*, forming several small sub-branches (**Figure 4**). This indicates that *S. bicolor* is evolutionarily related to *O. sativa* and *Z. mays* in the *CBL* gene family and may share some functions with them. For instance, SbCOBL5, SbCOBL9, SbECOBL5, and SbECOBL9 clustered with OsBC1L1 and OsBC1L8, as well as ZmBK2L1 and ZmBK2L8, forming a sub-branch with some maize CBL genes (**Figure 4**).

By comparing the conserved motifs of *CBL* genes in different species, it was found that several species have Motif1, Motif2, Motif3, and Motif4 (**Figure 3**). These motifs are found in CBL proteins from *S. bicolor*, *A. thaliana*, *O. sativa* and *Z. mays*. This suggests that they play important roles in the basic functions of the CBL gene family. They may be involved in the interaction of CBL proteins with other molecules or in maintaining the proteins’ structural stability. A comparative analysis of the CBL gene family with *CBL* gene families of other species reveals the evolutionary conservation and specificity of the *S. bicolor CBL* gene family. This conservation is reflected in similarities with other plants in terms of gene structure, conserved motifs, and evolutionary relationships. These similarities provide important clues for a deeper understanding of the CBL gene family’s basic functions.

### 4.3 Relationship between gene expression patterns and S. bicolor growth and development

Gene expression pattern analysis revealed that members of the *S. bicolor CBL* gene family exhibit distinct expression patterns in various tissues and developmental stages (**Figure 8**). Root growth and development is a complex process involving cell division, elongation, and differentiation, and *CBL* genes may affect cell morphogenesis and physiological functions by regulating cell wall synthesis and modification in root cells (Shao et al., 2020). In *A. thaliana* and *O. sativa*, it has been shown that *CBL* genes regulate the initiation and growth of root hairs (Roudier et al., 2005; Ben et al., 2015; Li et al., 2022). In root tissues, the high expression of *SbCBL1*, *SbCBL5* and *SbCBL9* may regulate root growth and development (**Figure 8**). For instance, these genes may regulate the orientation of cellulose microfilaments, affecting root cell elongation and differentiation. The highly expressed *CBL* genes in *S. bicolor* root tissues may have similar functions and play an important role in regulating root growth and development.

*SbCBL2*, *SbCBL4*, and *SbCBL8* are highly expressed in stem tissues (**Figure 8**). The mechanical strength of stems is crucial for upright growth and material transportation, as they are an important support structure in plants (Zhao et al., 2020; Zhang et al., 2022). *SbCBL2*, *SbCBL4*, and *SbCBL8* may enhance cell wall strength and toughness by regulating cellulose microfilament arrangement and deposition in the stem, thus promoting elongation and thickening. Phylogenetic analyses confirmed this hypothesis, showing that ZmBk2 maintains plant organ flexibility by regulating the interaction between lignin and cellulose (Sindhu et al., 2007). ZmBk2 clusters with SbECBL4 and SbCBL4, suggesting that it may perform similar functions through conserved molecular mechanisms. Different *SbCBL* genes showed differential expression patterns under drought and salt stress conditions (**Figure 9**). Specifically, NaCl treatment significantly increased the expression level of *SbCBL4* while decreasing the expression of *SbCBL3*, *SbCBL5*, *SbCBL7*, *SbCBL8* and *SbCBL9*. Conversely PEG treatment increased the expression of *SbCBL4* and *SbCBL8* while decreasing the expression of *SbCBL1*, *SbCBL2*, *SbCBL3*, *SbCBL5*, and *SbCBL9*. Analysis of the cis-acting elements of the *SbCBL* promoters revealed that *SbCBL5*, *SbCBL7*, *SbCBL8* and *SbCBL9* contain regulatory elements related to drought response. These results suggest that the *SbCBL* gene family plays a critical role in sorghum’s response to drought and salt stress. This role has also been confirmed in other plants, such as rice and cotton (Sun et al., 2022; Fu et al., 2024). This finding is consistent with previous research indicating that *CBL* genes play important roles in drought and salt tolerance in other species. These findings provide important clues for understanding the molecular regulatory mechanisms of *S. bicolor* growth and development and potential gene targets for genetic improvement and molecular breeding of *S. bicolor*. Future studies can explore these genes’ specific mechanisms during *S. bicolor* growth and development through gene function validation experiments. This will provide stronger theoretical support for improving *S. bicolor* varieties and agricultural production.

### 4.4 Limitations and prospects of the study

In this study, a more comprehensive bioinformatics analysis of the *CBL* gene family of *S. bicolor* was conducted. However, certain limitations still exist. First, regarding gene function validation, this study only analyzed gene structure, evolution, and expression patterns using bioinformatics methods. Thus, experimental validation of gene functions has not yet been carried out. Although potential gene functions were hypothesized based on gene structure and expression patterns, these hypotheses must be verified using techniques such as gene editing and transgenesis. Second, due to limitations in experimental conditions and technical capabilities, this study only used transcriptomic data for gene expression analysis, lacking protein-level validation. It is important to note that gene expression ultimately reflects protein expression; however, transcriptome data only reflects changes in the transcription level of genes, which does not accurately reflect protein expression. Future research can be carried out in the following directions: 1. To verify gene function, use CRISPR-Cas9 and other gene-editing technologies to construct *S. bicolor CBL* gene family knockout mutants and overexpression plants. Through phenotyping and analyzing physiological and biochemical indexes, study the specific functions and mechanisms of the genes in *S. bicolor* growth, development, and response to adverse stress. 2. Combine proteome data with transcriptome data to study protein expression. Using proteomics technology, we will analyze protein expression in different *S. bicolor* tissues and developmental stages. This will allow us to verify the relationship between gene and protein expression and further clarify gene function.3. We will study the interactions between members of the *CBL* gene family and other genes. Then, we will construct a gene regulatory network to help us understand the molecular regulatory mechanisms of *S. bicolor* growth, development, and response to adversity.

## 5 Conclusions

A total of ten *CBL* genes were identified in the genomes of BTx623 and E048 S. bicolor. Phylogenetic analysis revealed that *CBLs* can be classified into three subfamilies: Group I, Group II, and Group III. Gene duplication events indicate that WGD was the primary driver of the expansion of the *CBL* gene family. The tissue-specific expression patterns of *SbCBL* genes suggest varying expression levels across different organs and tissues in *S. bicolor*, with *SbCBL1*, *SbCBL5*, and *SbCBL9* showing significantly higher expression levels in roots. Furthermore, treatments with PEG and NaCl markedly affected the expression levels of *SbCBL* genes; specifically, *SbCBL4* expression increased following PEG treatment, while *SbCBL9* expression decreased after NaCl treatment. Overall, this study provides valuable insights into the role of the *CBL* gene family in *S. bicolor*.

## Data Availability

The datasets presented in this study can be found in online repositories. The names of the repository/repositories and accession number(s) can be found in the article/**Supplementary Material**.
